# Supplementary figures and images for: Interspecies interactions alter the antibiotic sensitivity of Pseudomonas aeruginosa
Source: Microbiol Spectr. 2024 Nov 4;12(12):e02012-24. doi: 10.1128/spectrum.02012-24 (PMC11619387; doi:10.1128/spectrum.02012-24)

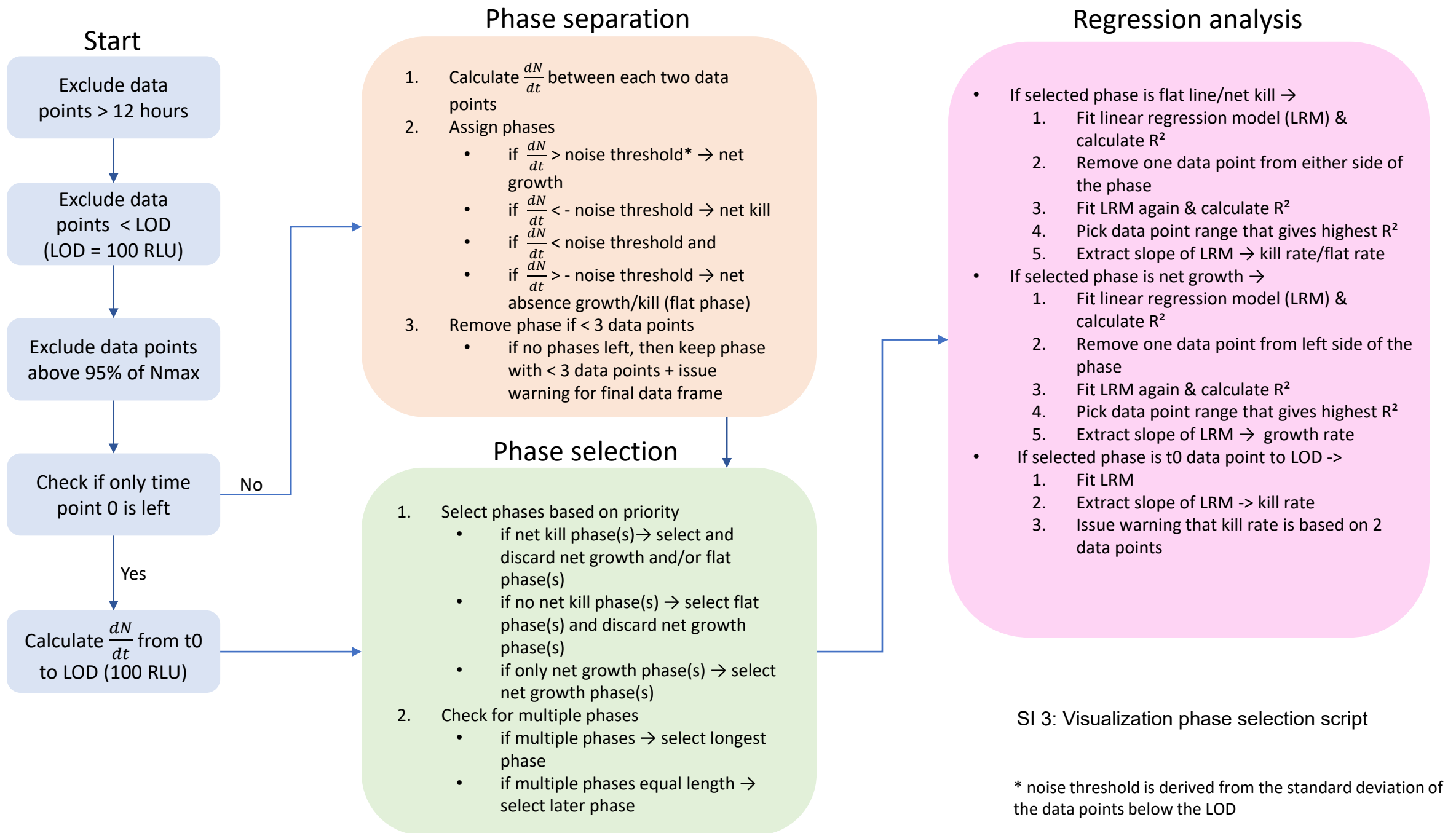

Supplement: SI 3 Visualization Phase Selection Script — Visualization of the phase selection script. [file spectrum.02012-24-s0003.pdf]

AZT

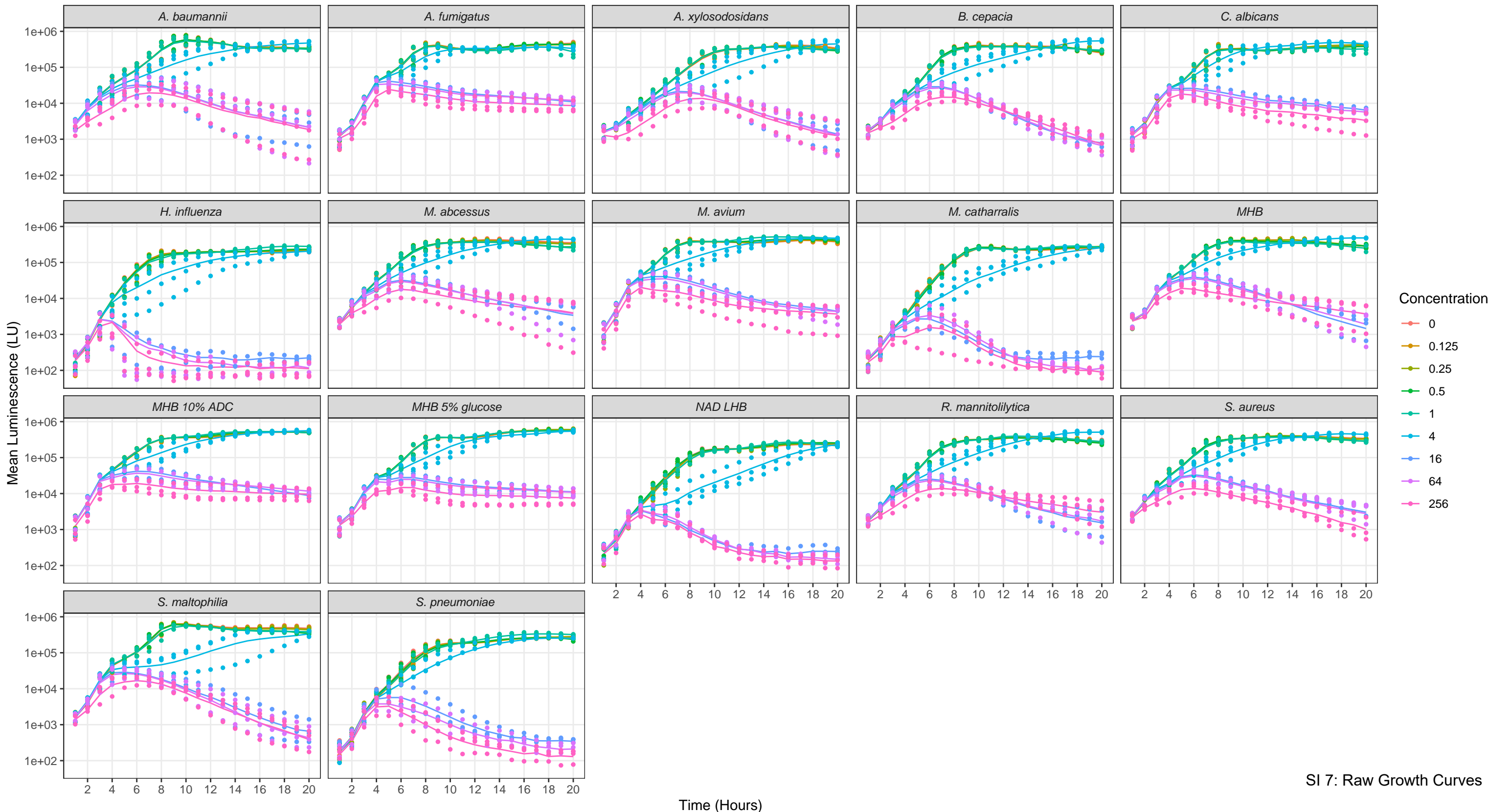

CEF

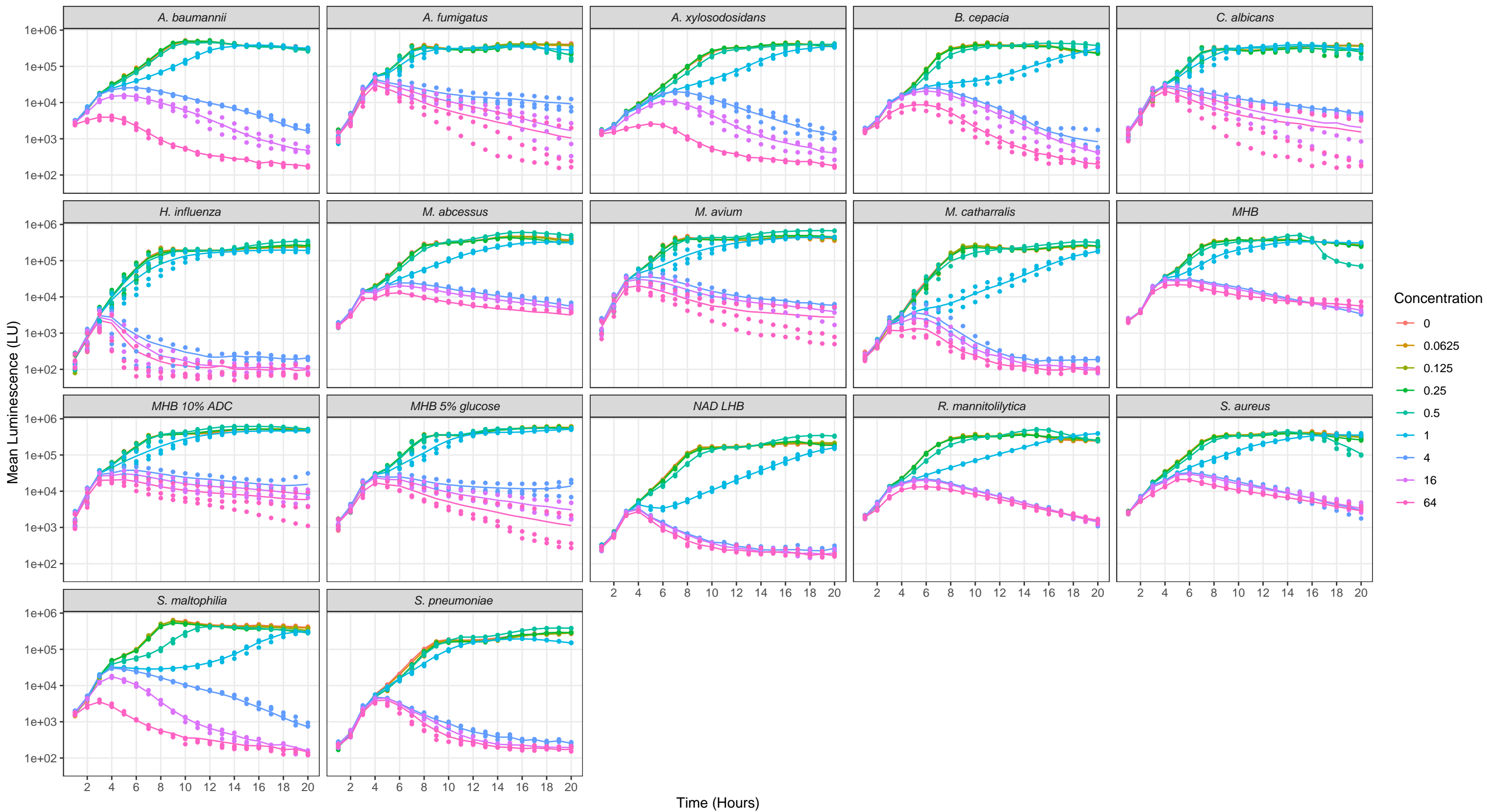

CIP

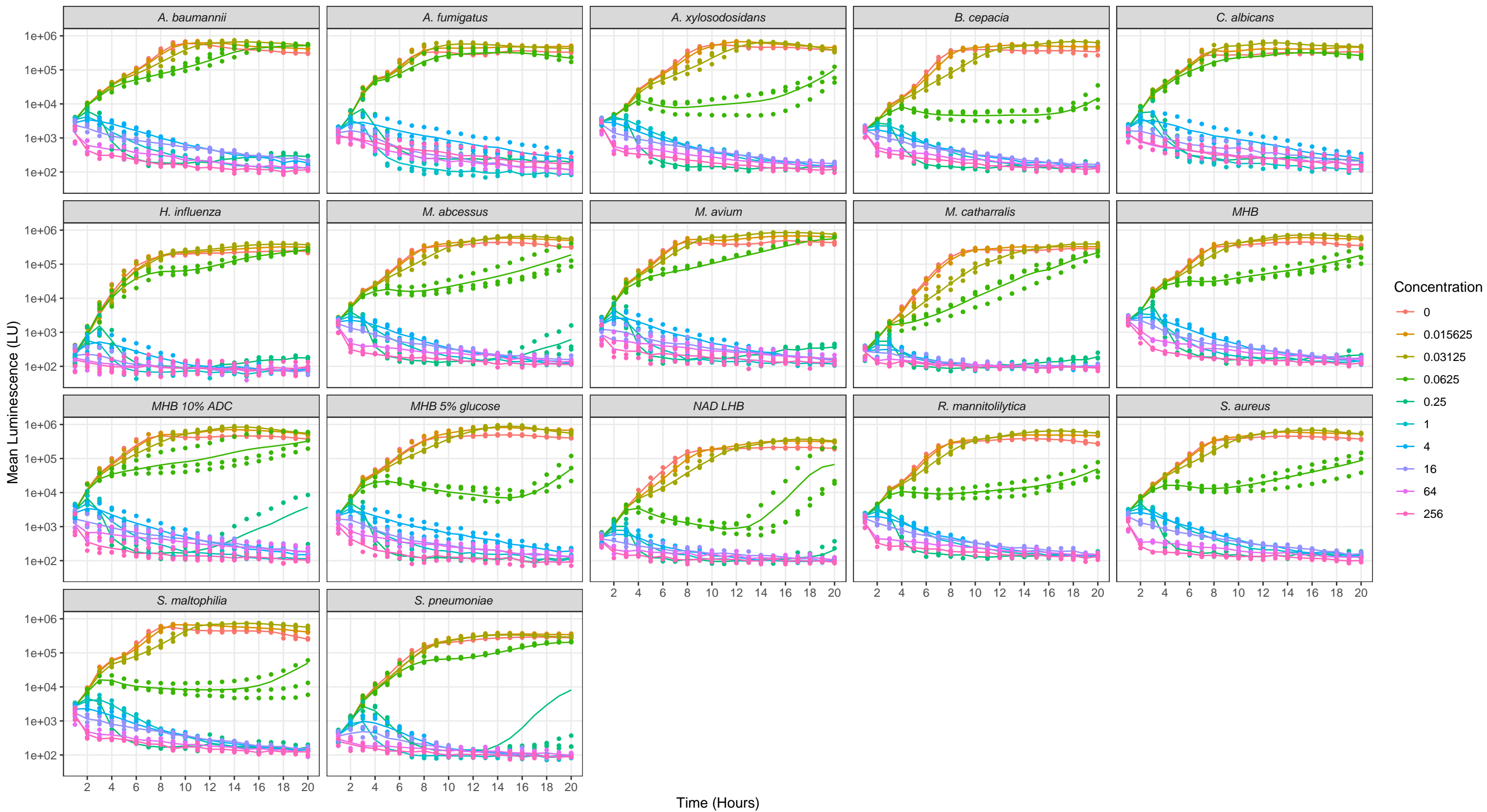

COL

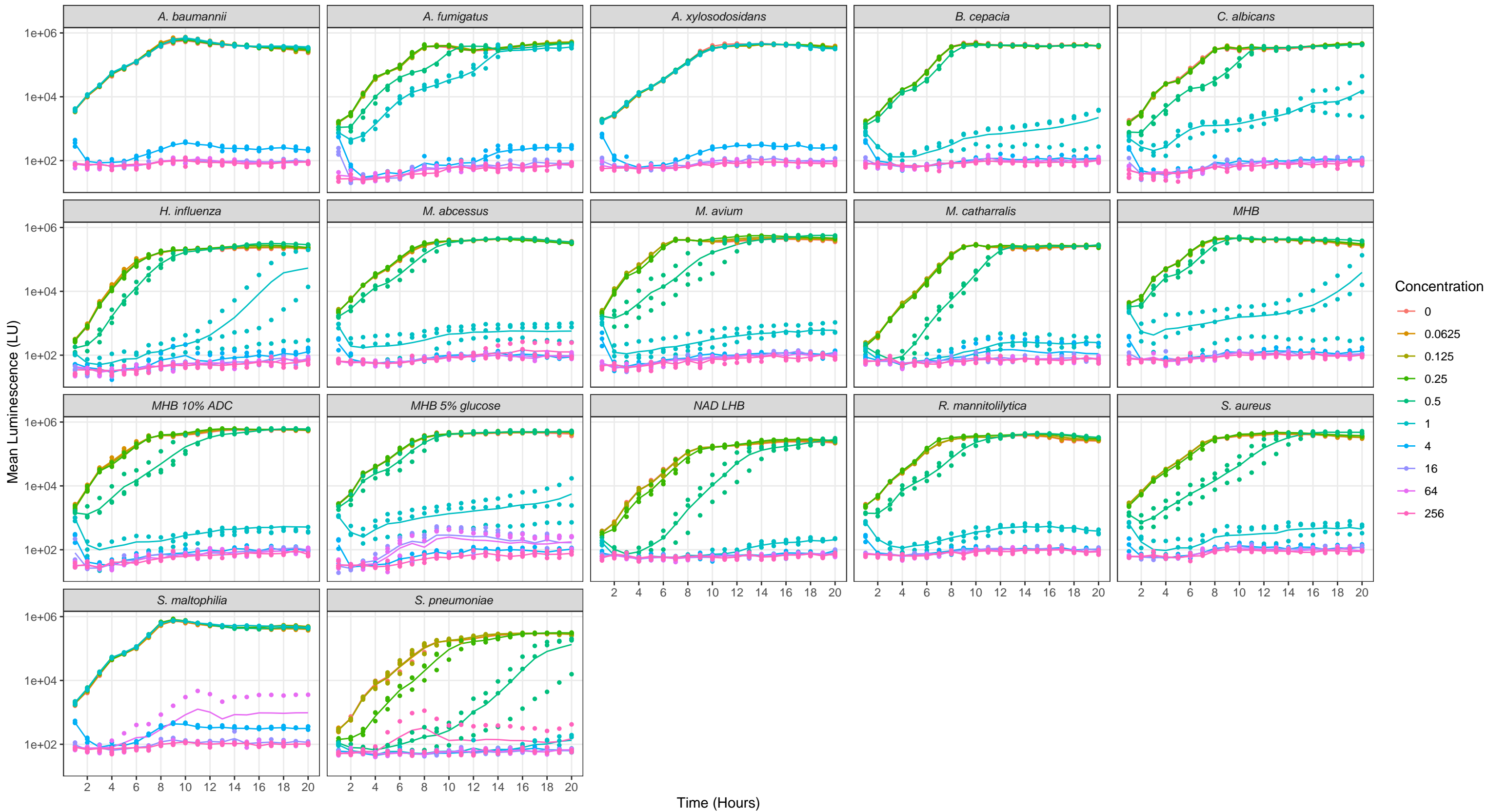

FOS

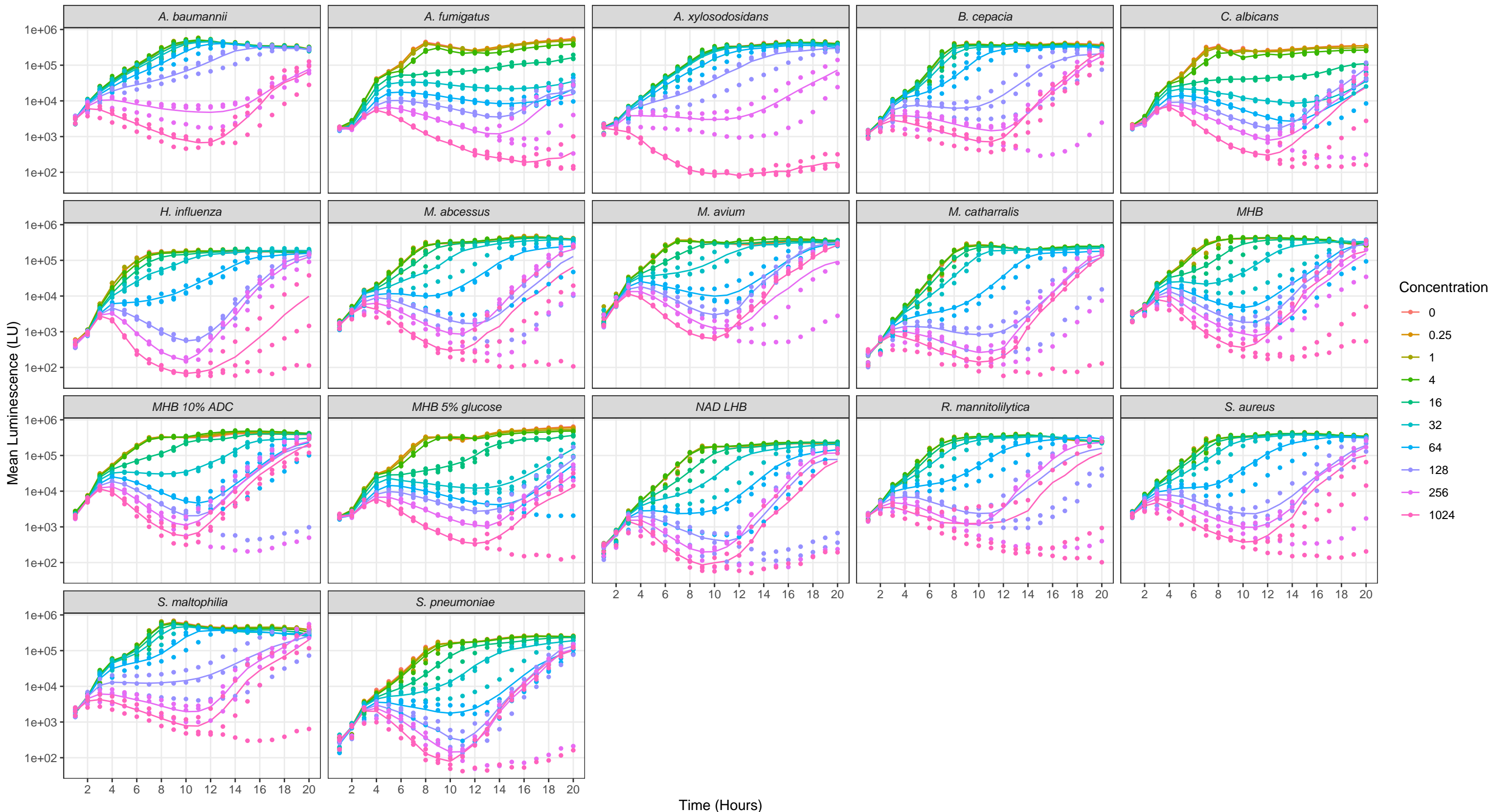

MER

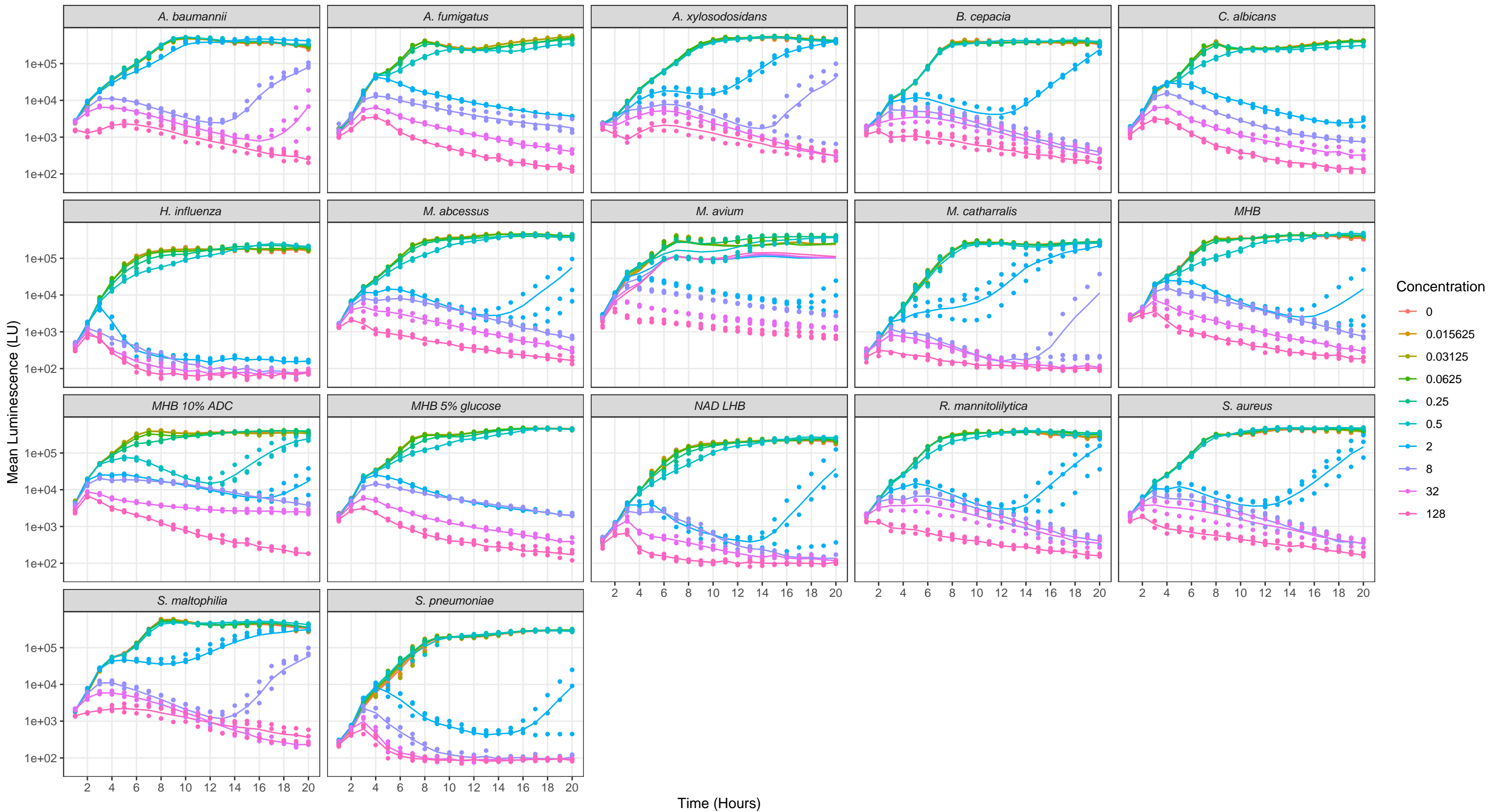

MIN

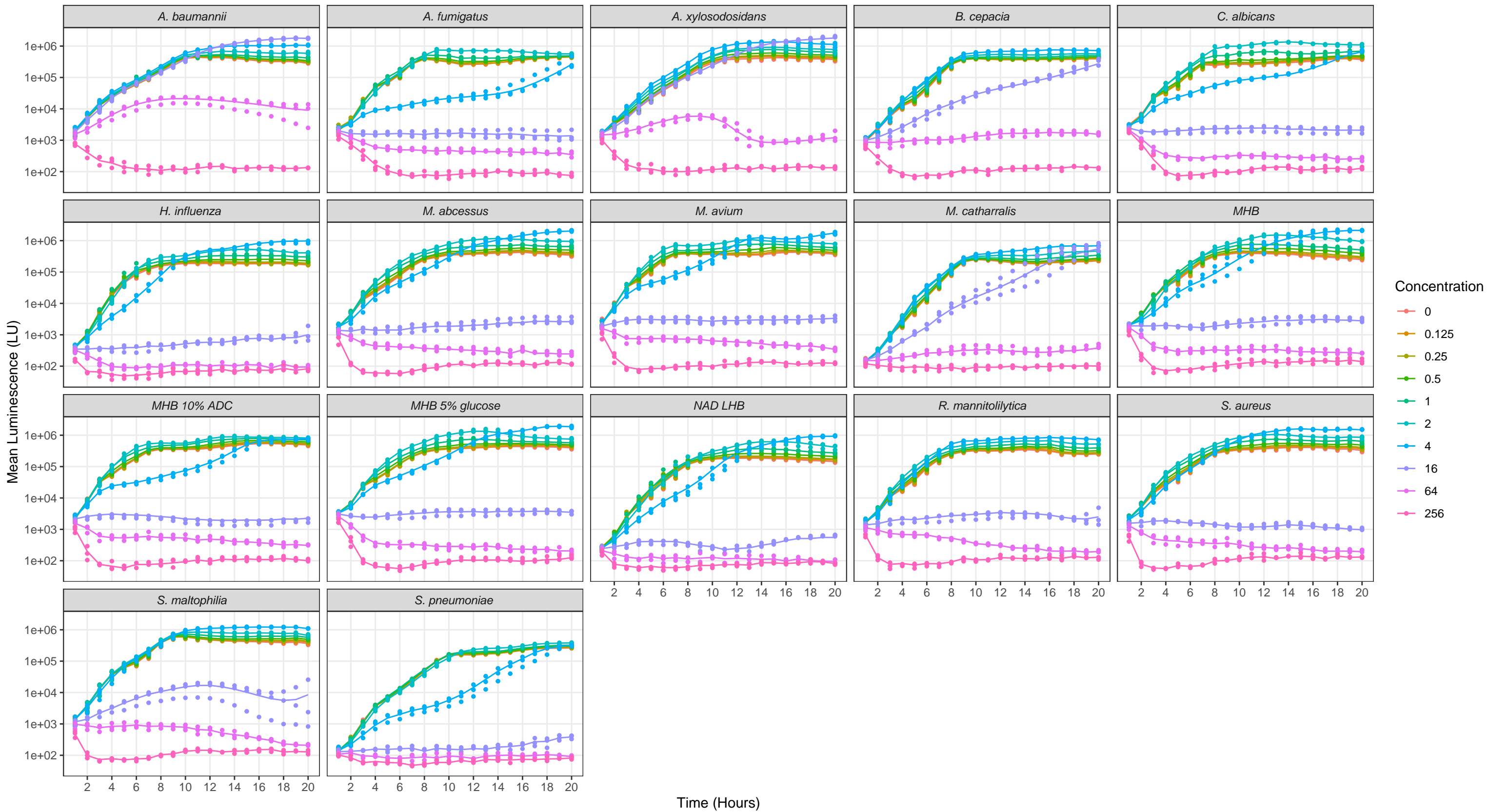

RIF

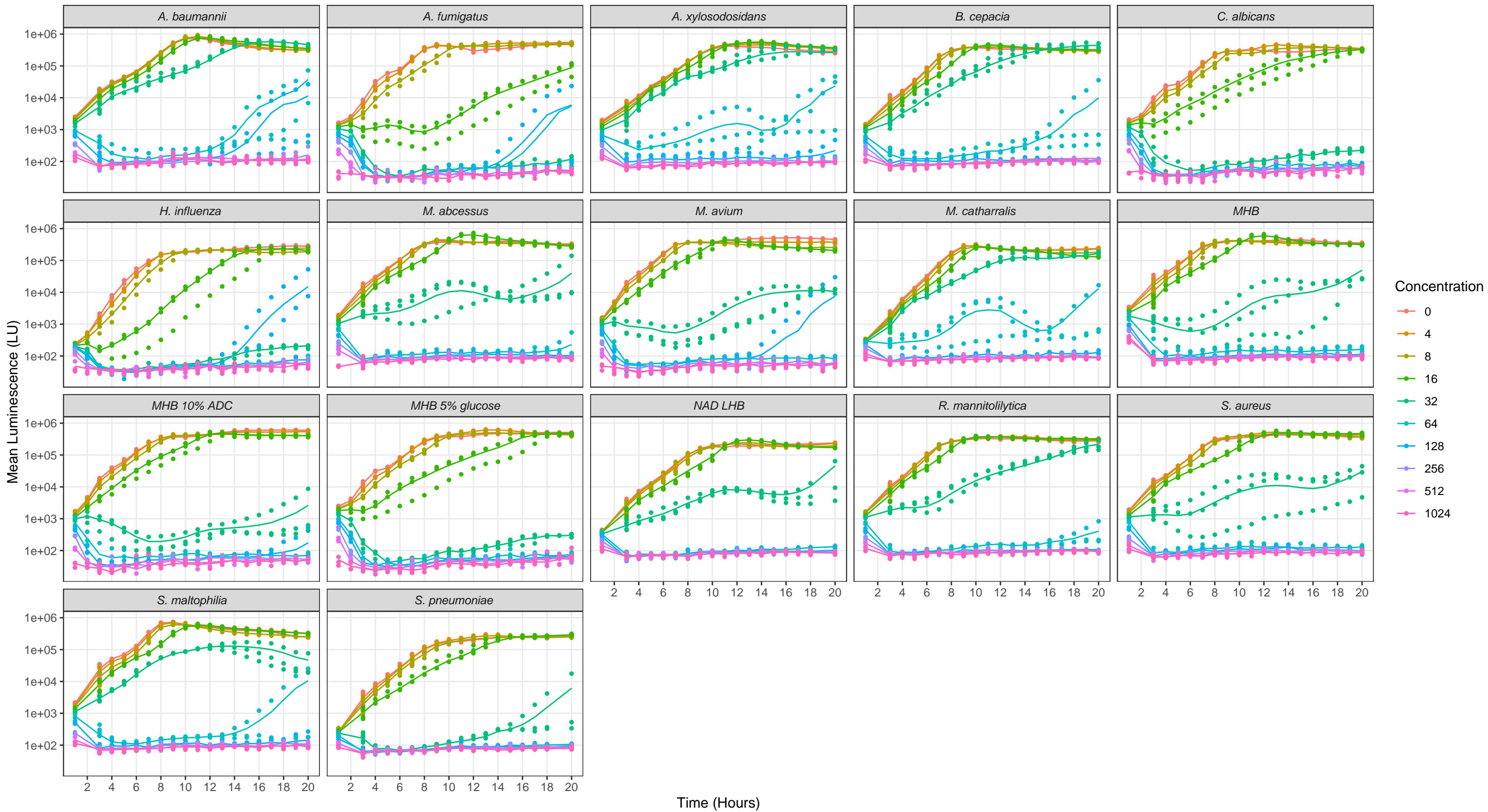

TOB

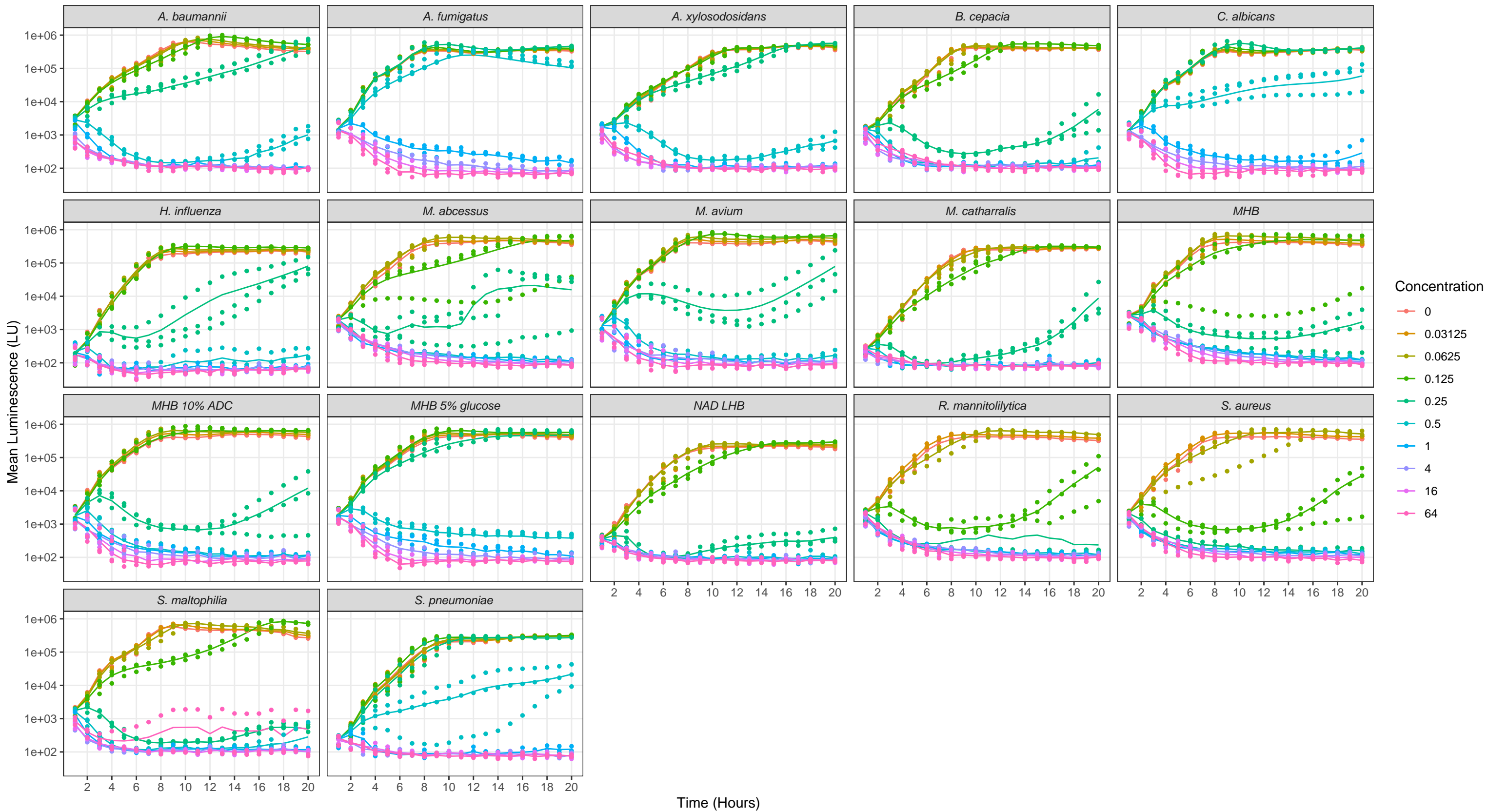

Supplement: SI 7 Raw Growth Curves — Plots with overview of all growth/kill curves. [file spectrum.02012-24-s0007.pdf]

AZT

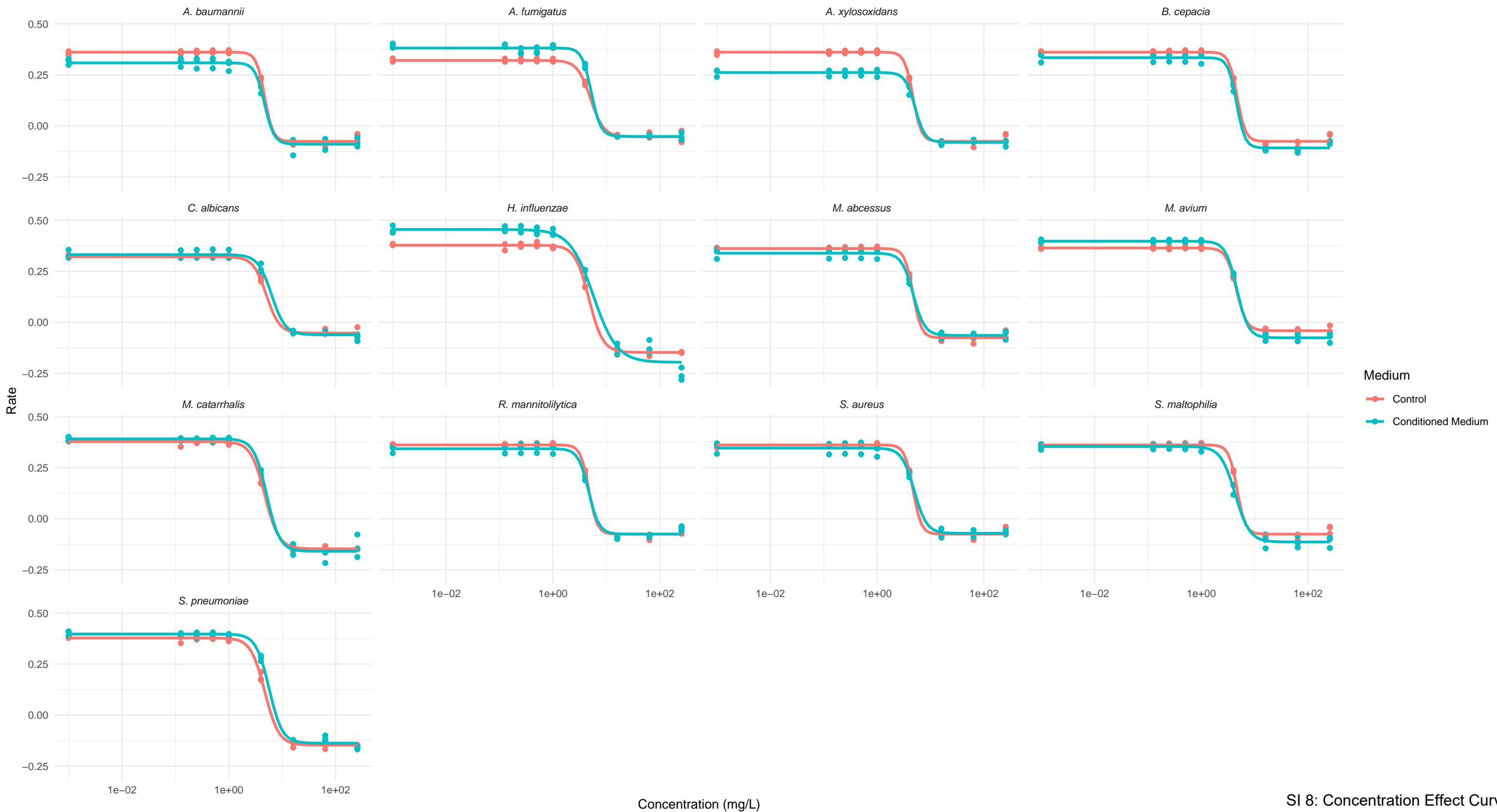

# CEF

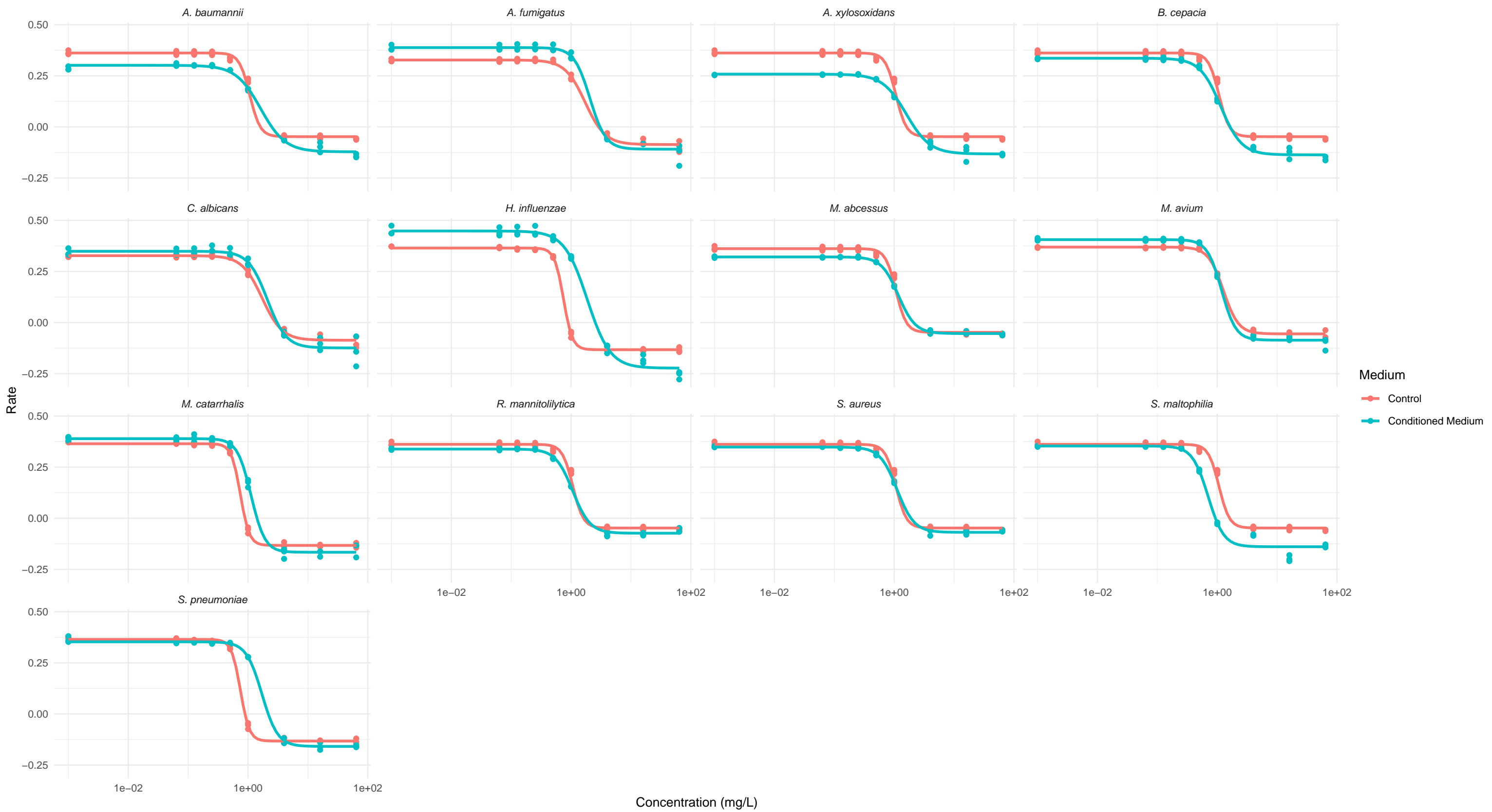

## CIP

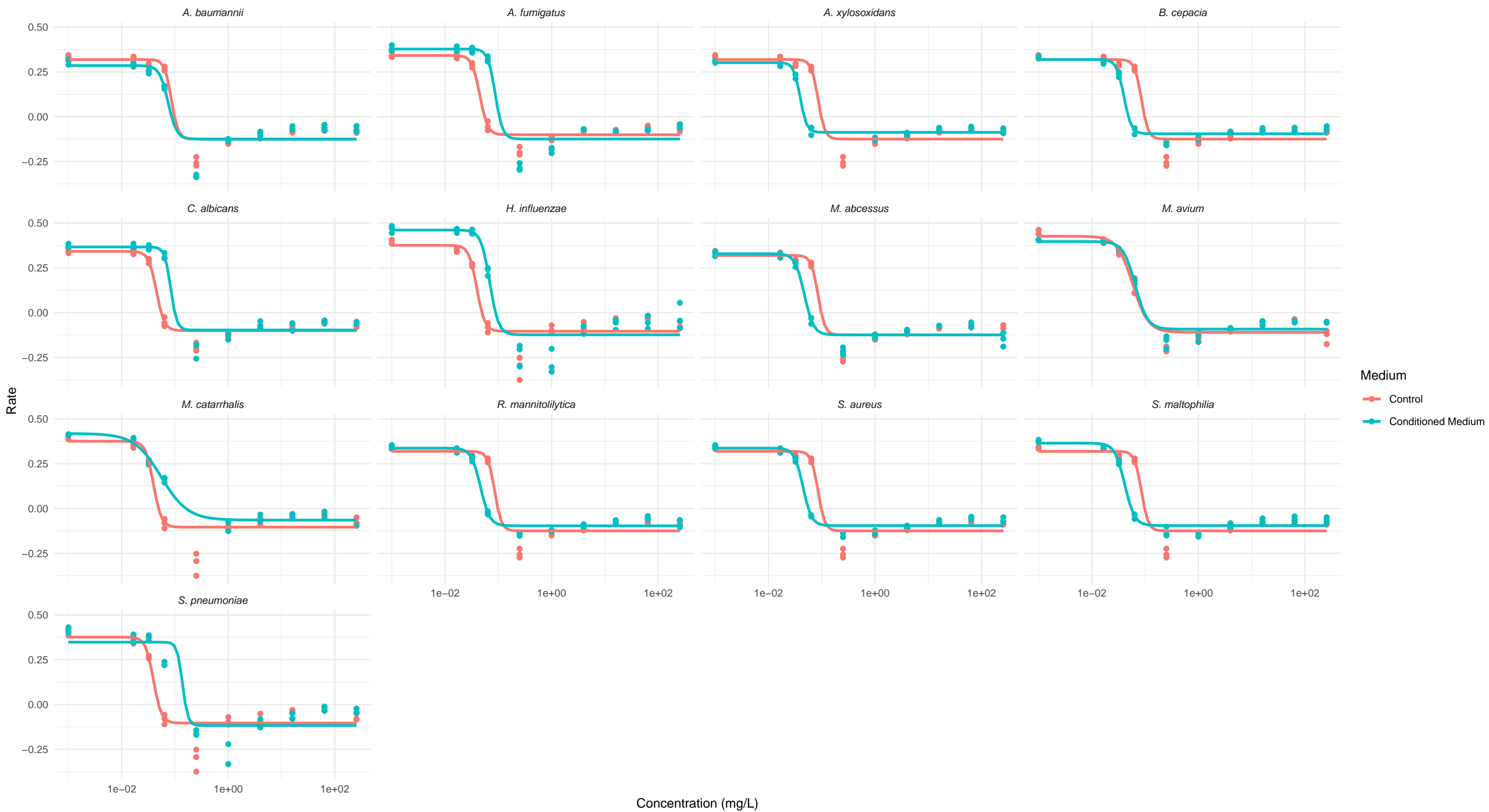

COL

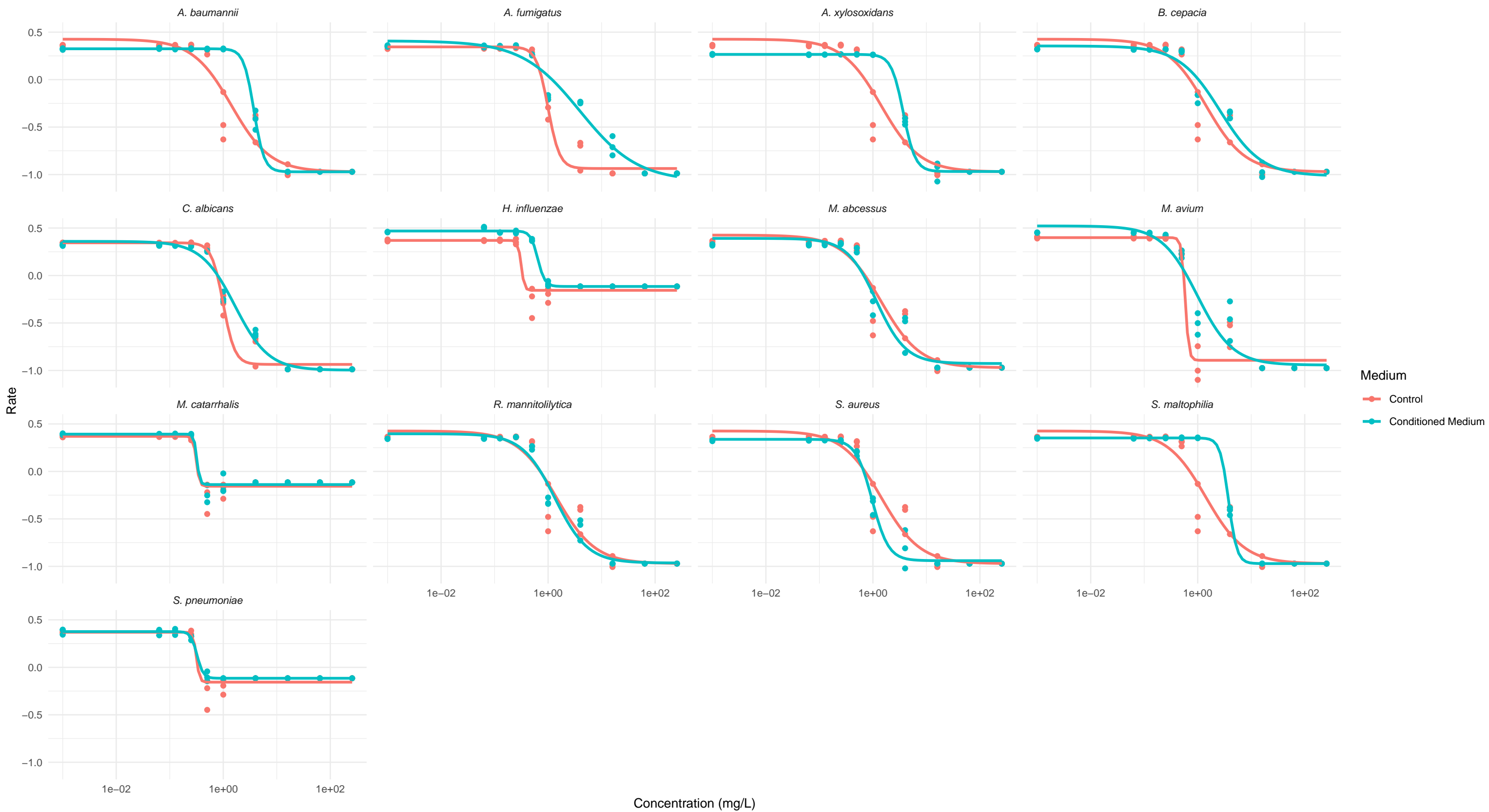

FOS

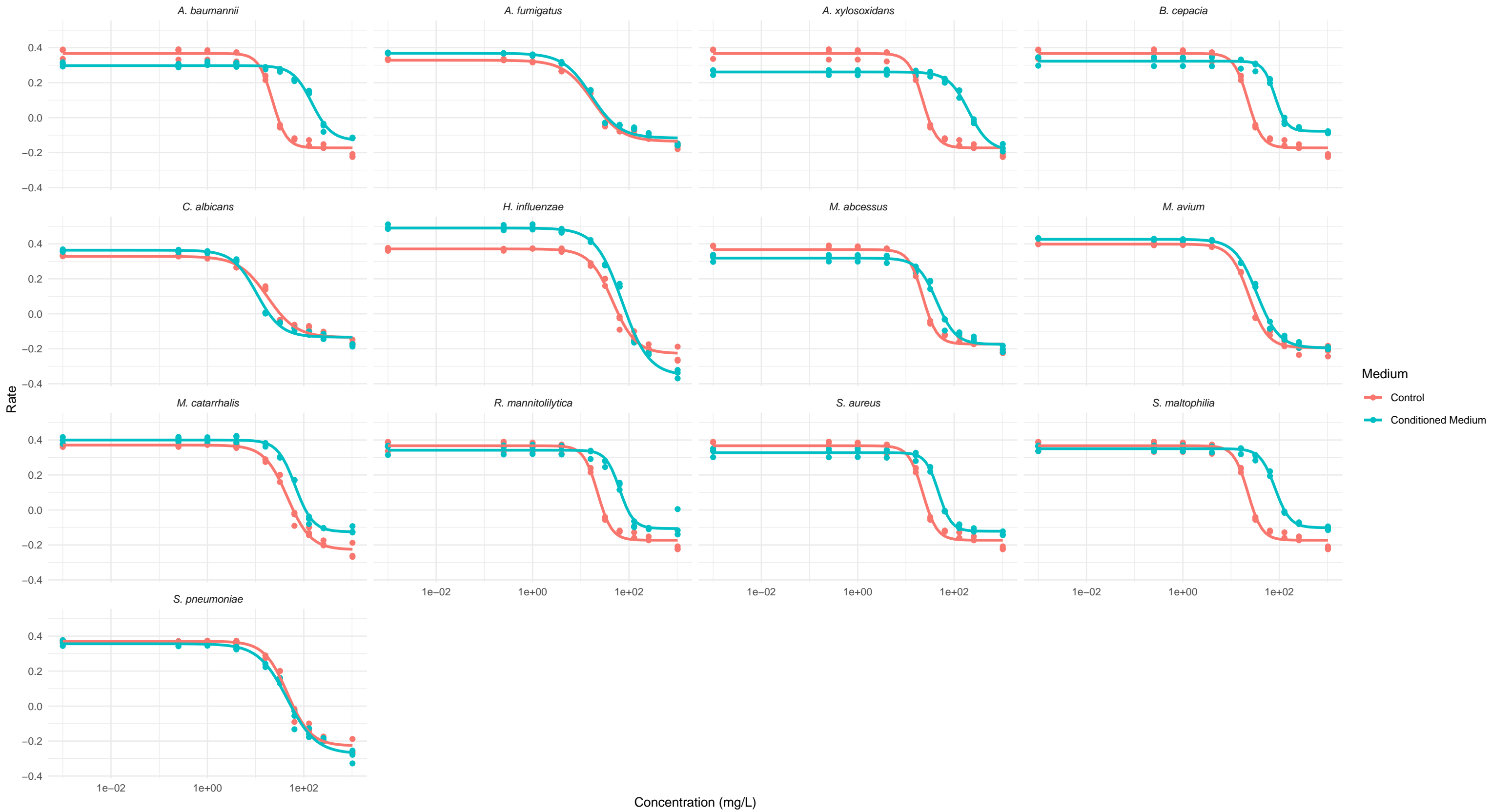

MER

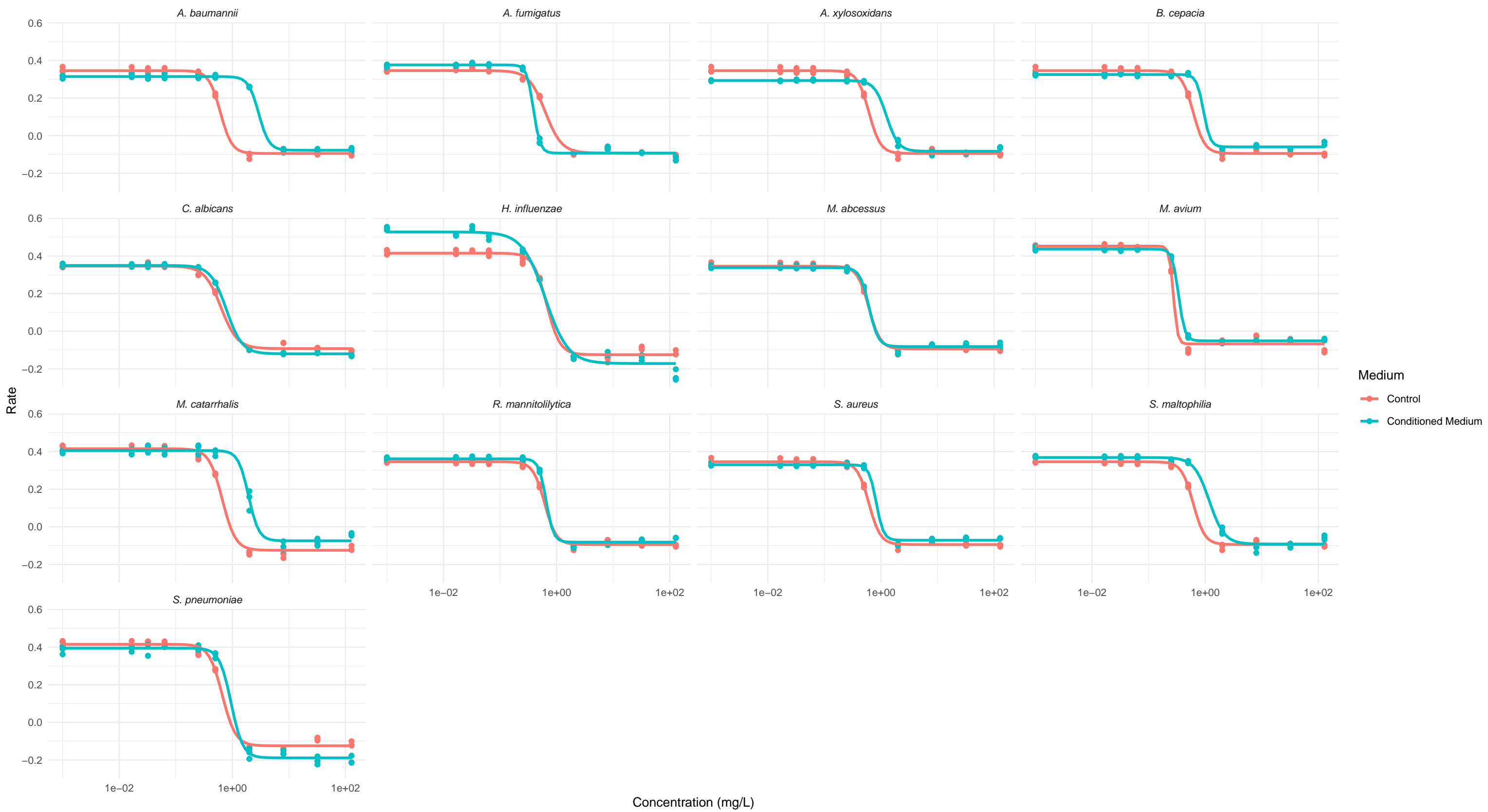

MIN

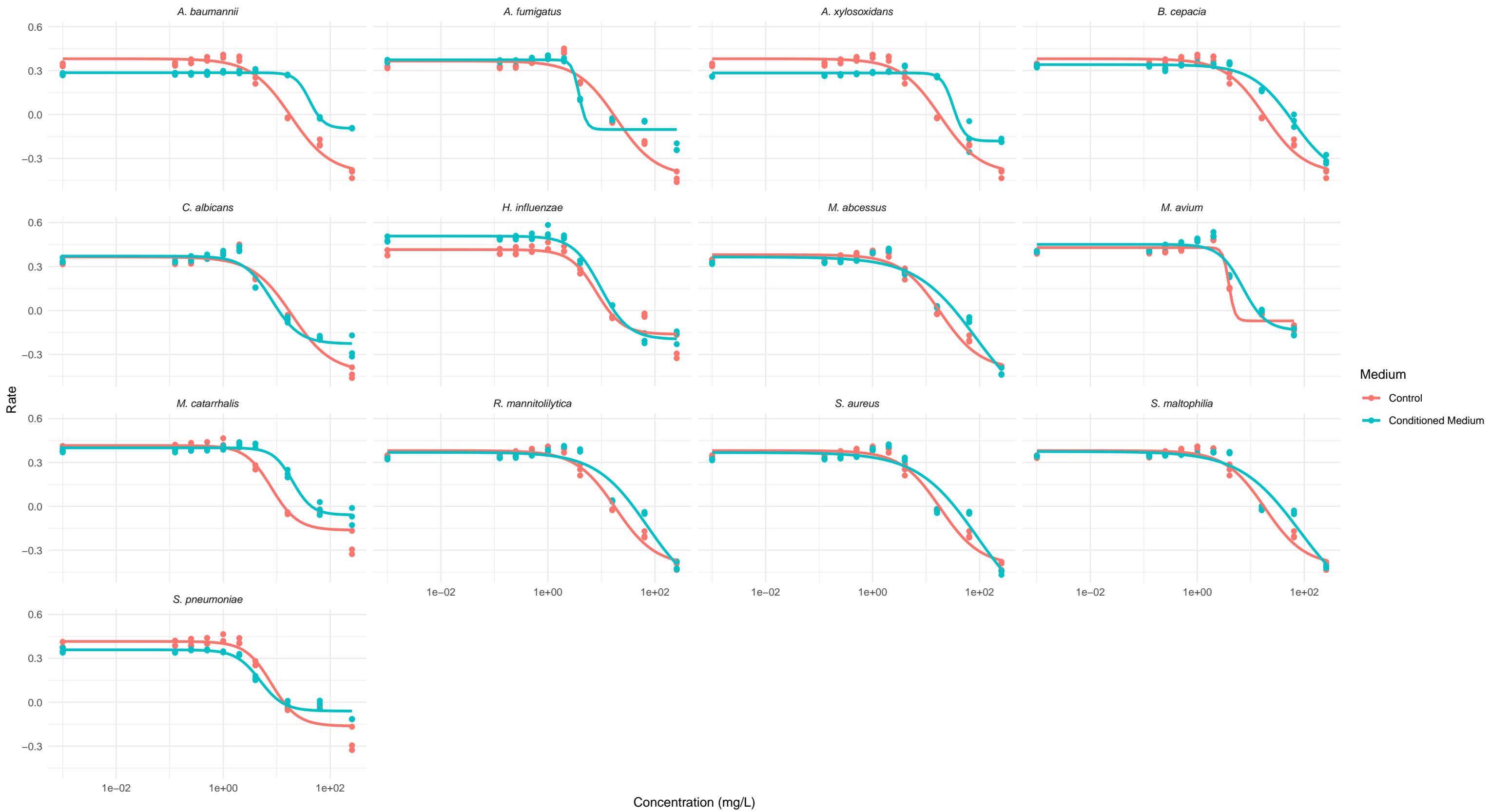

RIF

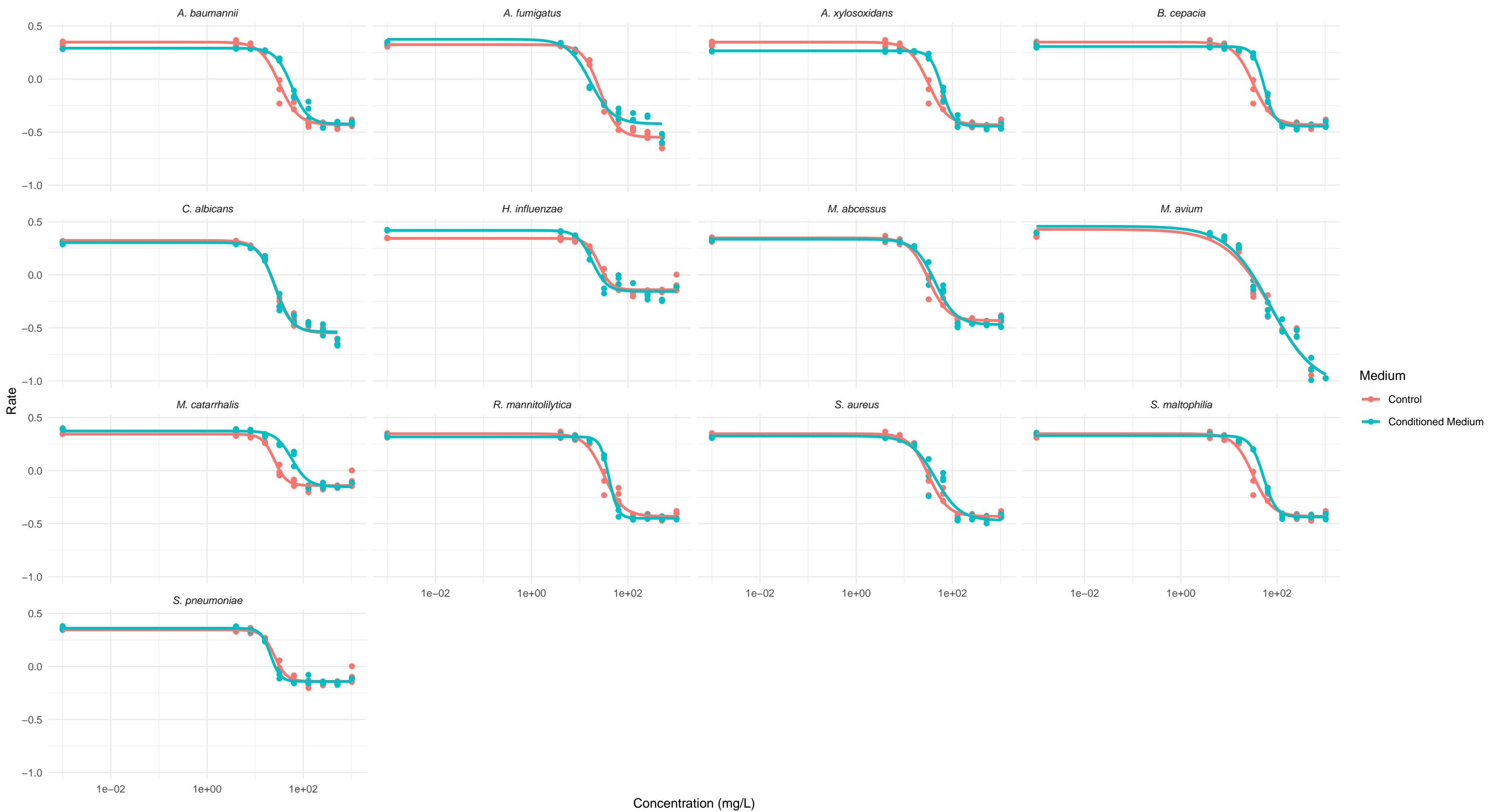

TOB

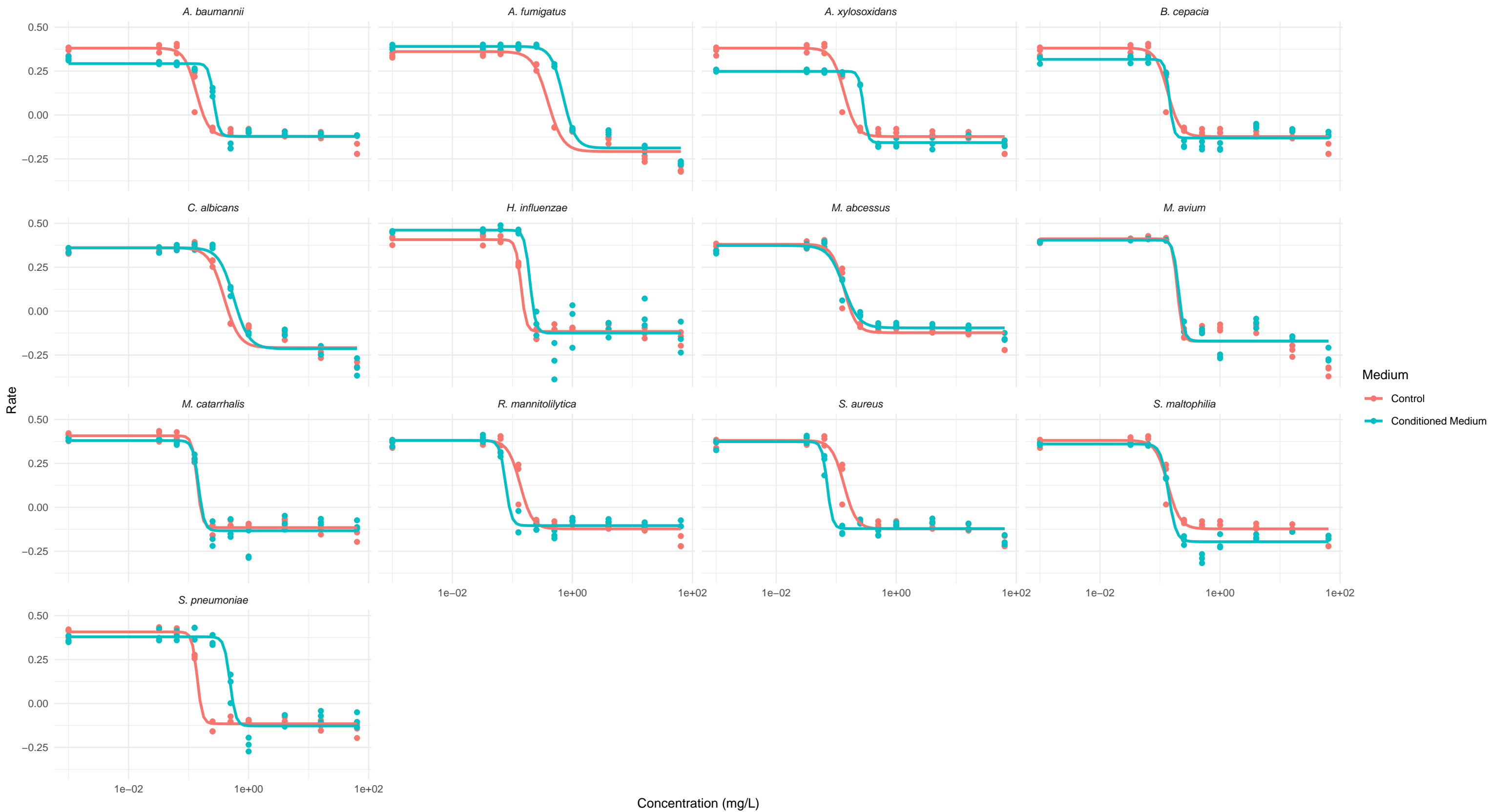

Supplement: SI 8 Concentration Effect Curves — Plots with overview of all determined concentration–effect curves. [file spectrum.02012-24-s0008.pdf]

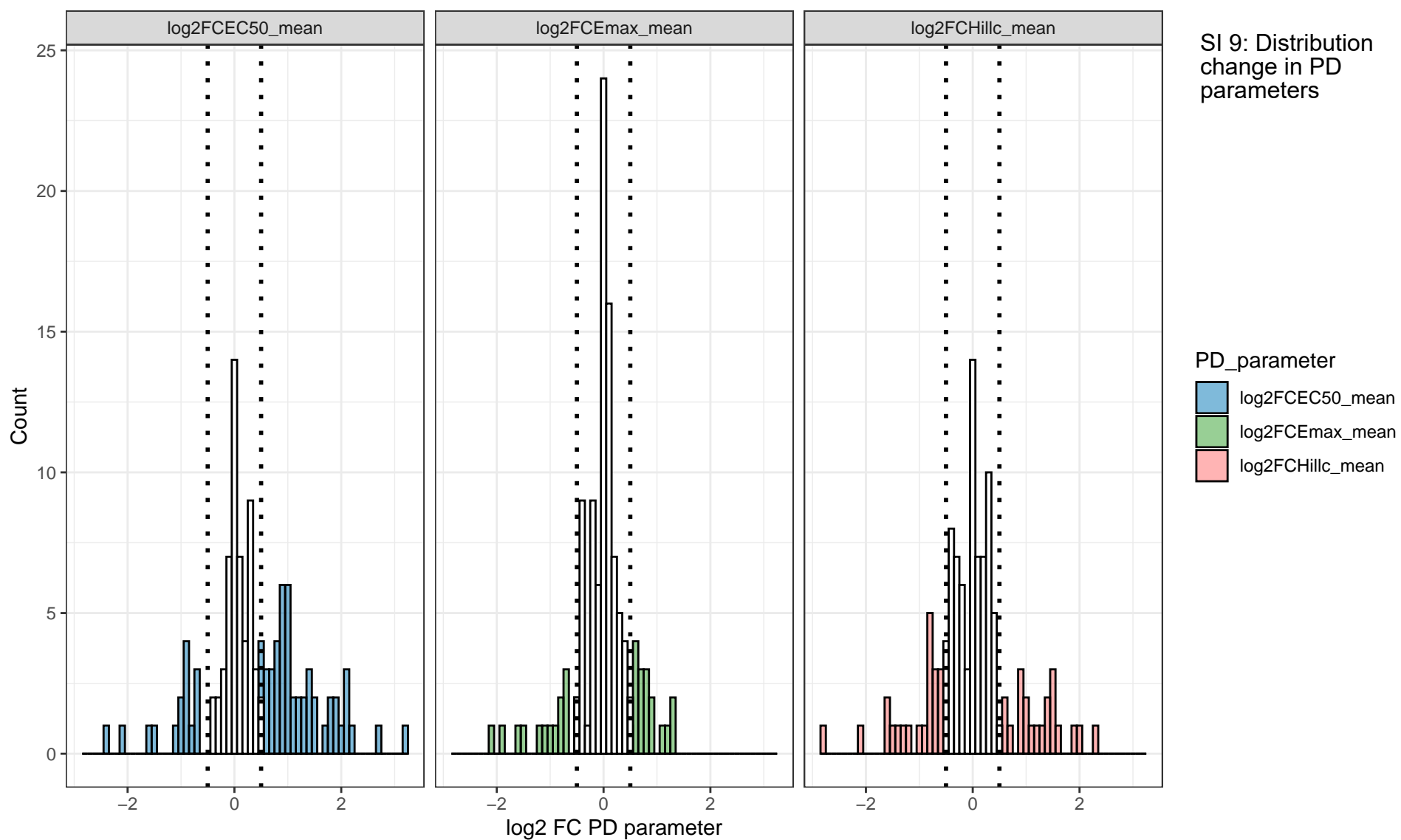

Supplement: SI 9 Distribution of PD parameters — Figure showing the distribution of the changes in PD parameters. [file spectrum.02012-24-s0009.pdf]
