# Supplementary material for: Interspecies interactions alter the antibiotic sensitivity of Pseudomonas aeruginosa
Source: Microbiol Spectr. 2024 Nov 4;12(12):e02012-24. doi: 10.1128/spectrum.02012-24 (PMC11619387; doi:10.1128/spectrum.02012-24)
Supplement: SI 10 LUM CFU Calibration Curves — LUM CFU calibration curves mentioned in the rebuttal. [file spectrum.02012-24-s0010.pdf]

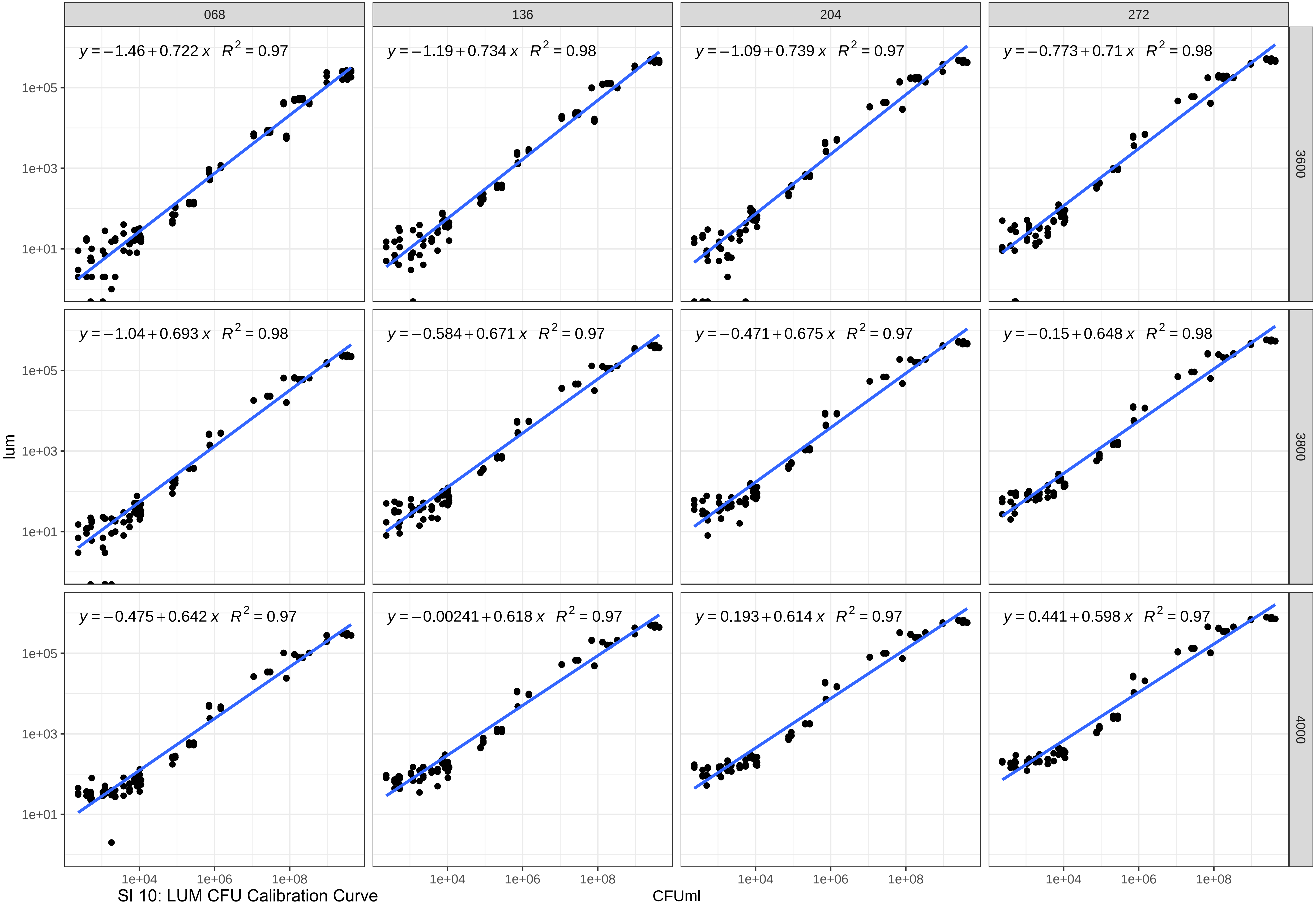

SI 10: LUM CFU Calibration Curve
